# Natural Killer Cell Responses in Hepatocellular Carcinoma: Implications for Novel Immunotherapeutic Approaches

**DOI:** 10.3390/cancers12040926

**Published:** 2020-04-09

**Authors:** Stefania Mantovani, Barbara Oliviero, Stefania Varchetta, Dalila Mele, Mario U. Mondelli

**Affiliations:** 1 Division of Infectious Diseases and Immunology, Department of Medical Sciences and Infectious Diseases, Fondazione IRCCS Policlinico San Matteo, 27100 Pavia, Italy; s.mantovani@smatteo.pv.it (S.M.); b.oliviero@smatteo.pv.it (B.O.); s.varchetta@smatteo.pv.it (S.V.); d.mele@smatteo.pv.it (D.M.); 2 Department of Internal Medicine and Therapeutics, University of Pavia, 27100 Pavia, Italy

**Keywords:** natural killer cells, hepatocellular carcinoma, NKG2D, MICA/B, immunotherapy

## Abstract

Hepatocellular carcinoma (HCC) still represents a significant complication of chronic liver disease, particularly when cirrhosis ensues. Current treatment options include surgery, loco-regional procedures and chemotherapy, according to specific clinical practice guidelines. Immunotherapy with check-point inhibitors, aimed at rescuing T-cells from exhaustion, has been applied as second-line therapy with limited and variable success. Natural killer (NK) cells are an essential component of innate immunity against cancer and changes in phenotype and function have been described in patients with HCC, who also show perturbations of NK activating receptor/ligand axes. Here we discuss the current status of NK cell treatment of HCC on the basis of existing evidence and ongoing clinical trials on adoptive transfer of autologous or allogeneic NK cells ex vivo or after activation with cytokines such as IL-15 and use of antibodies to target cell-expressed molecules to promote antibody-dependent cellular cytotoxicity (ADCC). To this end, bi-, tri- and tetra-specific killer cell engagers are being devised to improve NK cell recognition of tumor cells, circumventing tumor immune escape and efficiently targeting NK cells to tumors. Moreover, the exciting technique of chimeric antigen receptor (CAR)-engineered NK cells offers unique opportunities to create CAR-NK with multiple specificities along the experience gained with CAR-T cells with potentially less adverse effects.

## 1. Introduction

Hepatocellular carcinoma (HCC) accounts for approximately 90% of primary liver cancers and develops in a background of chronic viral hepatitis, alcoholic liver disease, or non-alcoholic steatohepatitis (NASH), after a multistep process requiring chronic inflammation leading to necrosis and cirrhosis. It is the second leading cause of cancer death and the fifth most common cancer worldwide [1]. HCC incidence is disproportionately increasing in men aged 55 to 64 years. 

HCC treatment options have considerably improved over the last few years, ranging from surgical resection, or loco-regional approaches (thermal ablation and transarterial chemoembolization, TACE), to liver transplantation or drugs such as sorafenib or lenvatinib for advanced disease and new second line options, including immune check-point inhibitors [2]. However, the overall HCC mortality rate remains disturbingly high. Despite the wealth of information on molecular biology, genomic and epigenomic, surveillance, diagnosis and management, there is currently a scarcity of seminal studies addressing the immunopathogenesis of HCC, which may have important implications in the design of immunotherapeutic strategies. Several studies point to the importance of innate and adaptive immunity in the control of cancer, including HCC. Natural killer (NK) cells, are an essential component of innate immunity, and changes in NK cell frequency and phenotype have been described during HCC development in a transgenic mouse model of aggressive human liver cancer [3]. Moreover, available evidence showed a positive correlation between the frequency of circulating and intrahepatic NK cells and survival in patients with HCC [4]. Interestingly, HCC cells express ligands of several activating NK receptors (NKR), including NKp30, natural killer receptor group 2, member D (NKG2D) and DNAM-1 such as the B7 protein homolog 6, the major histocompatibility complex class I chain-related protein A and B (MICA/B) and CD155, respectively, whose expression can correlate with the outcome of the disease [5,6]. Despite these findings supporting a role for NK cells in HCC immune surveillance, the pathogenetic mechanisms leading to HCC development and progression are poorly understood. Of note, functional deficiencies of circulating and intralesional NK cells have been demonstrated in various human cancers, including HCC [4,7,8].

Several studies support a role for NK cells and their activating receptor/ligand axes in HCC immune surveillance. Interestingly, patients with decreased expression of MICA on HCC tissue showed reduced disease-free and overall survival compared with patients with preserved MICA expression [9]. This finding strongly supports the involvement of the NKG2D receptor-MICA/B ligand axis (NKG2D-MICA/B) in NK cell-mediated tumor control. Other studies point to additional receptor-ligand axes, such as the DNAX Accessory Molecule-1 (DNAM-1) activating NKR and its ligand CD155, in HCC development [5]. Our recently published data point to an altered expression and function of the NKp30 activating receptor in circulating and resident NK cells of HCC patients, the former expressing an inappropriately high level of the Tim-3 exhaustion marker [6]. This, together with decreased expression of the major NKp30 ligand B7-H6 in liver cancer tissue relative to the stage of the disease suggests that this ligand play a major role in cancer surveillance. In addition, reduced expression of NKp30 immunostimulatory isoforms and increased expression of the inhibitory isoform in patients with advanced tumor, resulted in deficient NKp30-mediated functionality [6]. These findings provide compelling evidence in support of NK involvement in liver cancer immune control. In line with this, new approaches are being proposed for the treatment of tumors, such as the CAR-NK-based therapy (see below). Indeed, several phase 1 or 2 clinical trials for leukemia and myeloma as well as glioblastoma and non-small cell lung cancer are ongoing [10]. Moreover, a recent study [11] shows that a new type of NKG2D CAR-NK cells was able to delay disease progression of colorectal cancer in a mouse model and that their use in three patients with chemotherapy-refractory metastatic colorectal cancer produced antitumor effects.

Identification of additional relevant NK cell receptor/ligand axes may certainly represent an important target/source of information for immunotherapeutic interventions. Indeed, retrieval of the essential mechanistic insights involved in defective NK cell function will be instrumental for manipulation of the immune system to counteract tumor development and growth. Here we shall focus on the major receptor/ligand axis, i.e., NKG2D-MICA/B and on present and future applications of NK cells in immunotherapy.

## 2. Alteration of the NKG2D-MICA/B Axis in HCC

Among the activating NK receptor pathways mentioned above, NKG2D has received the attention of several investigators since it plays a pivotal role in NK cell responses toward modified or infected cells. NKG2D receptor is a hexameric complex consisting of two molecules of NKG2D expressed on the membrane cell associated each with two dimers of the signaling adaptor DNAX-associated protein 10 (DAP10) [12]. DAP10 molecules bring a Tyr-Ile-Asn-Met (YINM)cytoplasmic motif which is phosphorylated by Src family kinases after NKG2D engagement. The adaptor protein Grb2 and the regulatory subunit of phosphoinositide-3 kinase (PI3K), p85, associate with phosphorylated YINM motif determining the recruitment of guanine nucleotide exchange factor Vav1 and the activation of the kinase Akt, respectively [13], resulting in the cell activation.

This results in release of cytotoxic granules containing perforin and granzymes and cytokines such as Interferon γ (IFNγ), Granulocyte-Macrophage Colony-Stimulating Factor (GM-CSF) and macrophage inflammatory protein 1β (MIP-1β) with consequential killing of target cells and immunomodulation of other immune cell subsets, respectively [14,15]. The expression of NK receptors is tightly regulated by several factors, which in turn determine a modulation of NK cell responses. NKG2D expression can be modulated by up- or down-regulating cytokine-mediated stimuli [16]. Among the latter, in the context of tumor pathogenesis, transforming growth factor β (TGF-β) plays a pivotal role in the establishment of immune tolerance and control of inflammation via a suppressive action on innate and adaptive immunity consisting in the inhibition of expansion of NK and T cells and their cytotoxic and immunoregulatory functions [17,18]. On the other hand, these TGF-β mediated effects can facilitate the onset of tumors since they negatively impact on the quality of cancer immunosurveillance and anti-cancer response. In several solid and hematological malignancies, as well as in chronic hepatitis B and C virus infections, high levels of TGF-β have been associated with an impaired NK cell function and NKG2D expression [19]. Over the past several years, the mechanisms by which TGF-β down-modulates NKG2D expression have been shown to depend on both the induction of miRNA-1245, which directly controls NKG2D transcription [20], and down-regulation of DAP10 expression, resulting in a decreased NKG2D surface exposure [21]. Moreover, T regulatory cells (Treg) bearing TGF-β on their membrane can directly present it to NK cells determining reduced NKG2D expression [22]. Recently, a lot of interest focused on exosomes and microvesicles as vectors of a multitude of molecules potentially involved in the control of immune responses. In line with this, exosomes containing TGF-β derived from several tumor cell lines and platelets are able to induce NKG2D down-regulation [23].

The liver is an immunomodulatory organ populated by innate and adaptive cells and where the balance between the immune tolerance and activation toward antigens coming from the gastrointestinal tract is maintained by a peculiar microenvironment. TGF-β produced by liver sinusoidal endothelial cells and hepatic stellate cells is crucial in this regulation, contributing to induce Treg [24] with a consequent negative regulation of immune activity. However, due to its effect, it can become a tool for tumor cells to suppress the immune response, particularly targeting the NKG2D/NKG2DL axis. Such immunosuppression can occur in HCC progression where, particularly in the later stage, TGF-β facilitates cell invasion, angiogenesis, treatment resistance and transition of epithelial to mesenchymal cells [25]. TGF-β release can be linked to immunosuppressive cytokine interleukin-10 (IL-10) production in the HCC setting. Indeed, a B cell subset with immunosuppressive features has been found in HCC tissue, particularly in the advanced stage and in patients with poor survival. These Breg cells are characterized by expression of Tim-1 and by the release of IL-10 [26]. Also, in HCV-induced HCC, NS5A protein stimulates monocytes to produce the immunosuppressive cytokine interleukin-10 (IL-10) which can induce the release of TGF- resulting in impaired immune response [27].

Besides the various effects of TGF-β on NK cells functions, several other non-cellular components as well as cellular components of the tumor microenvironment may also have suppressive activities against NK cells, such as myeloid-derived suppressor cells, tumor-associated macrophages, cancer-associated fibroblasts. Moreover, the physical status of the HCC microenvironment, including characteristics such as hypoxia and the production of metabolites by the tumor cells may also impair NK cell function. For example, lactate, adenosine, indoleamine 2,3-dioxygenase can interfere with NK cell activation and phenotype [28,29,30].

In line with our preliminary unpublished data, Easom et al. [31] recently showed a decreased expression of NKG2D on NK cells derived from HCC tissue compared to matched uninvolved liver tissue. They showed that NKG2D down-modulation depended on contact between NK and HCC cells with consequent internalization of the receptor. These results are supported by a previous study by Quatrini et al. who showed that DAP10 ubiquitination occurred after receptor engagement by MICA but not by ULBP2, which provided a signal leading to NKG2D internalization and trafficking of the NKG2D/DAP10 complex from the membrane to lysosomes where it was degraded. Receptor-mediated endocytosis via ubiquitination was needed to activate the intracellular signaling pathway which resulted in NK cell activation [32].

In this context, the role of NKG2D ligand (NKG2DL) expression is critical for efficient binding to the receptor with consequent NK cell activation leading to an effective immune response. NKG2DLs comprise the UL16-binding proteins (ULBPs) and the MHC class I related proteins (MICA and MICB). To date, six ULBP (ULBP1-6) molecules encoded by genes clustered in human chromosome 6 are known. They contain α1 and α2 domains and can be transmembrane (ULBP4 and 5) or glycosylphosphatidylinositol (GPI)-linked (ULBP1-3, 6) or both (ULBP2 and 5) proteins [33]. MICA/B contain α1, α2 and α3 domains and are encoded by genes in the MHC [34]. They are transmembrane proteins, except for the MICA*008 allele which contains a mutation upstream of the transmembrane region leading to the expression of a truncated GPI-linked molecule [35].

It is well-established that the NKG2DL expression is upregulated in stressed, infected or cancer cells, thereby placing the physiological identification of target cells from immune cells by an “induced self-recognition” process [33,36]. As the NKG2D/NKG2DL axis plays a crucial role in the immune response, tumor cells have developed several escape mechanisms which regulate NKG2DL expression and which may have an impact on the interaction with NKG2D. For instance, virus-infected cells can control MICA/B gene expression, as in HBV infection, and this upregulates the transcription factors GATA-2 and GATA-3 which in turn suppress MICA/B expression by direct binding to the promoter region [37]. Moreover, Wen and colleagues [38] showed that HL7702 human hepatocytes transfected with plasmids encoding the HCV serine protease NS3/4A expressed decreased levels of MICA compared to control, whereas the UL148A-HCMV protein contributed to down-modulate MICA by targeting it for lysosomal degradation in HCMV-infected cells [39].

In the HCC setting, Kamimura and colleagues demonstrated that tumor progression and early recurrence correlated with loss of ULBP1 expression in HCC cells and that its regulation depended upon proteasome activity [40]. Again, hepatotropic viruses such as HBV can significantly down-regulate MICA expression, as shown for the HepG2.2.15 HBV-producing human hepatoblastoma cell line [41]. The evidence gathered was further supported by subsequent studies which showed that miRNA-induced by the major HBV envelope polypeptide inhibited the MICA/B expression in HCC cells [42] and that overexpression of the miRNA 25-93-106b cluster determined suppression of MICA in HCC cells [43]. Another tumor escape mechanism is NKG2DL shedding caused by several molecules belonging to the a disintegrin and metalloproteinase (ADAM) enzyme family whose activity is regulated by the phosphorylation of their cytoplasmatic tail by intracellular kinases such as polo like kinase 2, MAPK, and protein kinase C [44]. Recently, it has been shown that also changes in the cellular membrane symmetry due to cell activation and resulting in externalization of negatively charged phosphatidylserine lead to an increased sheddase activity of ADAM17 [45]. Among the substrates of ADAM proteases such as adhesion molecules, growth factors, cytokines and their receptors, MICA/B are found be the target of cleavage by ADAM10 and ADAM17 [46,47], whereas ULBP2 is cleaved by other metzincins [48]. In the HCC setting, it has been shown that ADAM9, which is overexpressed in HCC tissue, was involved in MICA shedding at the level of an intracellular cleavage resulting in the release of the soluble form of MICA [49]. Interestingly, decreased sMICA levels and increased membrane-bound MICA expression in HCC could be attained by ADAM17 knockdown [50], an enzymatic pathway which is also involved in the control of FcγRIII shedding in chronic HCV infection [51]. Regorafenib, a small molecule inhibitor of multiple kinases approved for treatment of sorafenib-resistant HCC, inhibited mRNA and protein expression of ADAM9 and ADAM10 in HCC cell lines, resulting in higher membrane MICA expression and conversely lower sMICA levels [52], similarly to the popular aldehyde dehydrogenase inhibitor disulfiram which suppresses ADAM10 activity [53]. The soluble forms of NKG2DLs block the activation of NKG2D pathway protecting tumor cells from NK cell-mediated cytotoxicity [54]. In line with this, we found an increased level of sMICA/B in sera of HCC patients with different etiologies (unpublished data). Moreover, high sMICA concentration were found in HCC patients with shorter overall survival [55]. In line with these findings, new therapeutic approaches based on blocking the ADAM activity including the use of low molecular weight synthetic inhibitors, purified or synthetic forms of ADAM pro-domains, modified tissue inhibitor of metalloproteinase 2 and monoclonal antibodies are been investigated [56]. 

NKG2DL release mediated by exosomes also represents an efficient HCC cell evasion system. Indeed, it has been demonstrated that MICA*008-containing exosomes can downmodulated NKG2D expression and inhibit NK cytotoxic effect [57,58]. Finally, post-transcriptional events can be involved in MICA/B downmodulation [59]. In fact, hypoxia, a cell stress stimulus which is often associated with HCC, can determine the accumulation of incorrectly folded and incorrectly glycosylated newly synthesized proteins in ER, resulting in the activation of unfolded protein response (UPR). This is associated with MICA/B proteasomal degradation and consequently reduced NK cell cytotoxicity [60].

**Figure 1:**
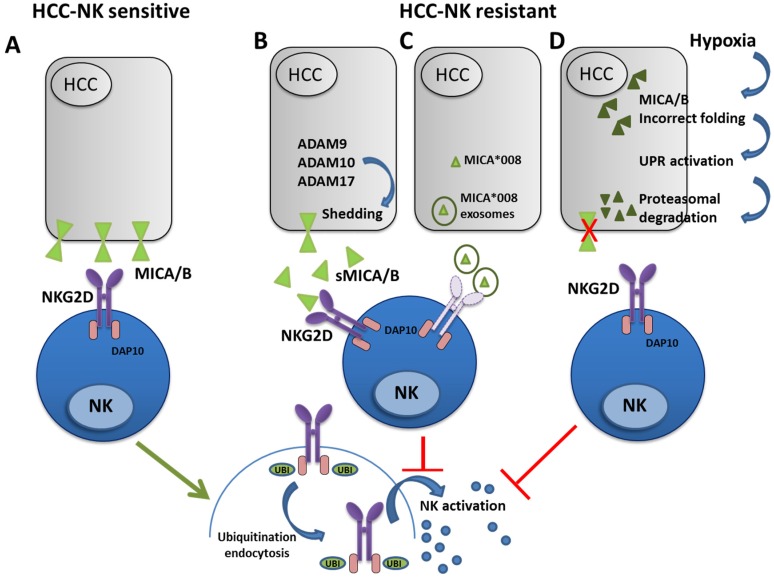
Hepatocellular carcinoma (HCC)-dependent alterations of the NKG2D-MICA/B axis. MICA/B molecules expressed on HCC cells are recognized by NKG2D receptor making them HCC-Natural Killer (NK) sensitive. This induces ubiquitination (UBI)-mediated endocytosis of NKG2D-DAP10 complex leading to NK activation and consequent HCC killing (**A**). In the right panels several mechanisms of evasion resulting in HCC being NK resistant are illustrated. (**B)** sheddases ADAM9, 10 and 17 cleave MICA/B molecules producing the soluble form (sMICA/B) which binds NKG2D receptor. This blocks binding to membrane MICA/B with consequent NKG2D-mediated NK activation. (**C**) MICA release mediated by exosomes can downmodulate NKG2D and inhibit the NK cytotoxic effect. (**D**) UPR-dependent proteasomal degradation of unfolded MICA/B proteins results in its reduced membrane expression. These mechanisms disrupt NK cell activation and anti-tumor response.

Thus, all the evidence gathered so far point to the importance of dysregulation of the NKG2D/NKG2DL axis in tumor cell escape and growth, suggesting that restoration of a correct immune surveillance may represent a novel immunotherapeutic approach for HCC (Figure 1).

## 3. NK-Based Immunotherapy

**Figure 2:**
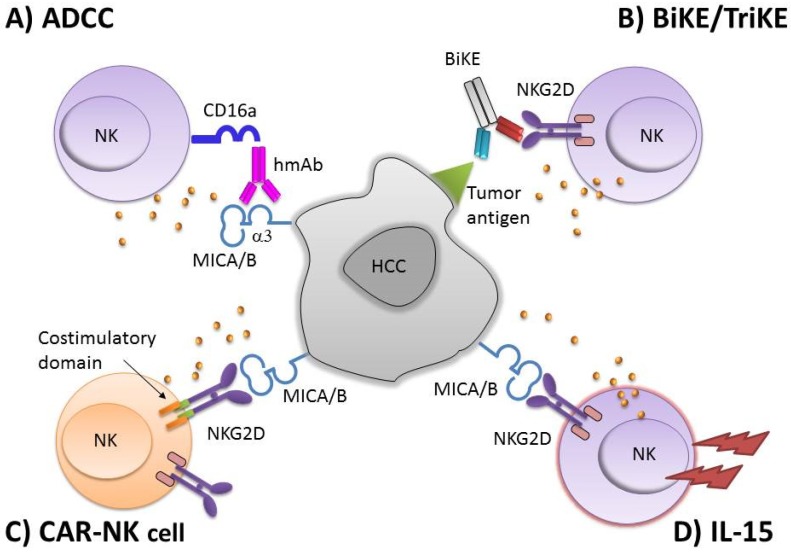
Possible NKG2D-MICA/B-based immunotherapy approaches. Different strategies can be used to induce NK cell activation to improve HCC killing. (**A**) MICA/B α3 domain–specific mAb is able to induce NK cell-mediated antibody-dependent cytotoxicity (ADCC). (**B**) Bi-specific or tri-specific killer engagers (BiKE or TriKE), capable of binding NKG2D expressed on NK cells and one tumor antigen on HCC can maximize targeting of tumor cells. (**C**) A chimeric antigen receptor containing the NKG2D extracellular domain that binds to MICA/B ligands, fused to a spacer and trans-membrane domain. The intracellular domain contains costimulatory domains, such as CD28 and 4-1BB and the CD3ζ chain, which drive signal activation and amplification of CAR-NK cells. (**D**) cytokines, such as interleukin (IL)-15, can also be employed to generate cytokine induced NK cells.

Over the past several years, there has been a growing interest in harnessing the NK anti-tumor ability as a novel immunotherapeutic approach and several clinical trials have been designed or are being planned based on NK cell infusion (Table 1). Some of these are in progress, including a phase I study (NCT03319459) in which the effect of allogeneic ex vivo activated NK cells is evaluated as monotherapy or in association with monoclonal antibodies (trastuzumab or cetuximab) in several advanced solid tumors, including HCC. Other trials are recruiting patients for use of allogeneic NK cells in support of drug therapies (NCT04162158) or ex vivo expanded autologous immune killer cells in combination with loco-regional procedures such as TACE (NCT03592706). Moreover, studies based on NK cell infusion are completed (Table 1), even if data are not yet available except for the phase III NCT00699816 trial in which adjuvant immunotherapy with activated autologous Cytokine-induced killer (CIK) cells in patients undergoing curative treatment for HCC resulted in increased recurrence-free and overall survival [61]. Recently, IL-15 has been evaluated in clinical trials as potential support for immunotherapy because of its activating effect on NK cells but not on Treg cells. Unfortunately, despite improvement in NK cell expansion, no durable responses have been obtained [62]. In contrast to other cytokines that are secreted, IL-15 primarily exists bound to the high affinity IL-15Rα. When IL-15/IL-15Rα complexes are conveyed to the cell membrane, they can stimulate opposing cells through the β/γC receptor complex. This novel mechanism of IL-15 delivery has been named trans-presentation. Therefore, the increased biological activity of trans-presented IL-15 has been exploited to improve IL-15 mediated NK cell efficiency. Several methods to mimic trans-presentation in vivo have been evaluated [63], including infusion of IL-15Rα-Fc pre-ligated to recombinant IL-15 (IL-15/IL-15Rα) in mice and in human xenograft models in which an expansion of NK cells, as well as naïve and memory CD4 and CD8 T cells was obtained. Another complex has been generated by directly binding IL-15 to the IL-15Rα “sushi+” domain, the region resulting in high-affinity binding of trans-presented IL-15 to IL-15R [64]. This technique allowed boosting of effector NK and CD8+ T cells in vivo (murine, human xenograft and primate models) showing a higher response toward metastatic melanoma and colorectal tumors [65]. Another fusion protein, obtained by coupling an IL-15 superagonist mutant (IL-15N72D) to an IL-15Rα/IgG1-Fc (ALT-803 by Altor Bioscience Corporation), exhibited superior immunostimulatory activity, prolonged in vivo pharmacokinetics and increased in vivo biologic activity against B cell lymphoma and glioblastoma compared to IL-15 in mouse models [66,67]. In the HCC setting, data from mouse models are encouraging, as shown by the inhibitory effect exerted on HCC by an NK cell line transfected with IL-15 [68]. In line with these and other findings recently obtained by Easom and colleagues [31] who showed that IL-15 could restore NK cell activity in HCC, it is possible to envision an immunotherapeutic approach by harnessing NK cell activity with IL-15 for HCC (Figure 2).

## 4. NKG2D Based CAR-NK Cells

One encouraging branch of cancer immunotherapy is CAR-engineered immune cells. The CAR is an artificially modified fusion protein which combines an extracellular antigen recognition domain, followed by a spacer and transmembrane region and fused to a wide range of intracellular signaling domains. The ectodomain of CARs is a single chain variable fragment (scFv) that recognizes a specific antigen, typically overexpressed on or exclusive to tumor cells, that is not presented in the context of major histocompatibility complex (MHC) molecules, similarly to an antibody. The intracellular domains of the second and third-generation CARs usually include, in addition to CD3ζ, one or two costimulatory signaling molecules, such as 4-1BB (CD137), CD28, CD27, OX40 (CD134), inducible T cell co-stimulator (ICOS) or regulatory subunit I anchoring disruptor (RIAD) that extend the strength of signal afforded by the CAR, enhance survival, cell activation, proliferation, cytokine secretion and cytotoxicity against tumor cells that express the CAR-specific antigen [69]. The solid-tumor microenvironment is immunosuppressive and an obstacle for all immunotherapies, including CAR therapies [70]. The “next-generation” CARs are principally designed for treatment of heterogeneous solid tumors, engineered to deliver immunomodulatory cytokines to shape the tumor microenvironment, to improve migration with the insertion of chemokine receptors such as CXCR1 or CXCR2 and to include suicide genes to improve safety. Other approaches are aimed at improving the ability to evade immunosuppression by including in the structure additional domains that either limit suppressive signaling or convert suppressive signals into activating signals [70,71,72].

Over the past few years, research on CAR has largely focused on T cells (CAR-T) [73]. Notably, two CD19-CAR-T products have gained commercial approval for the treatment of relapsed and refractory pre-B cell acute lymphoblastic leukemia and for diffuse large B-cell lymphoma by the U.S. Food and Drug Administration as the first gene therapy [74,75]. However, the risk of cytokine release syndrome and/or neurologic toxicities as well as the costly and labor-intensive approach to personalize the treatment reduce their clinical applications [76,77]. CAR-modified NK cells have attracted much attention as an alternative to CAR-T cells as NK cells intrinsically lack these shortcomings. At present, no CAR-NK cell product has received marketing authorization, but several phase 1-2 clinical trials are ongoing in hematologic and solid tumors. NK cells derived from NK cell lines, donor or autologous peripheral blood mononuclear cell (PBMC) and umbilical cord blood have been modified to express CARs against different cancer targets, including CD7, CD19, CD22, CD33 for lymphoma and leukemia, HER2 for glioblastoma, MUC1 for HCC (NCT02839954) and NKG2D for metastatic solid tumors (NCT03415100) [10,78,79]. Importantly, NK cells express activating receptors, such as NCRs, NKG2D, and DNAM-1 which are rapidly triggered by engagement of ligands expressed by stressed and transformed cells [80]. The naturally occurring cytotoxicity mediated by NK cells can complement CAR-induced cell killing and may allow CAR-NK cells to bypass the loss of targeted antigens as a tumor escape mechanism [81]. Moreover, ADCC is an additional NK cell-mediated tumor-killing strategy [82] that could increment CAR antitumor activity.

Most clinical CAR-NK therapies derive directly from the engineering structures used in the CAR-T cell field. Indeed, Chang et al. designed a chimeric receptor termed NKG2D-DAP10- CD3ζ to retrovirally transduce NK cells which became consistently more cytotoxic and produced more cytokines than mock-transduced cells against leukemia and solid tumor cell lines. Expression of a CAR in NK cells significantly augmented NK cell functional activity over the endogenous NKG2D receptor alone [83]. An innovative approach involves substitution of the CD3ζ region with an intracellular domain that is specifically involved in NK cell activation. Guo et al. employed the extracellular domain of the check-point inhibitory receptor PD-1 to reverse the immune escape mediated by PD-1 ligands in solid tumors. The authors designed a chimeric PD1-NKG2D receptor containing the NKG2D hinge region and the 4-1BB co-stimulatory domain to exhibit stable surface expression and mediate in vitro cytotoxicity exhibited by NK92 cells against tumor cells [84]. A second promising approach has been devised by Xiao and colleagues who fused the NKG2D extracellular domain to DNAX Activating Protein 12 (DAP12), as the intracellular component of CAR-NK. In order to address the concern of on-target and off-tumor toxicity of an NKG2D-CAR against non-tumor tissues, the authors adopted an RNA CAR approach to transiently enhance the specificity of NK cells toward NKG2DLs. The non-integrating mRNA electroporation technology was used to express CARs into cells. By means of short-lived CAR-expressing cells, the duration and potency of CAR effects can be controlled by different dosing and infusion schemes. The transient expression of CARs on immune cells requires multiple infusions to achieve antitumor effects but an excessive response caused by toxicity related to recognition of normal tissues and/or cytokine storms can be controlled. This study demonstrated that NKG2D-DAP12-CAR significantly augmented the cytolytic activity of NK cells against several solid tumor cell lines in vitro and provided a clear therapeutic benefit to mice with established solid tumors. Notably, the authors treated three patients with metastatic colorectal cancer with local infusion of RNA CAR-modified NK cells. In two patients, a significant reduction of ascites was obtained together with a marked decrease of tumor cells in the ascitic fluid after infusion of a single low dose of cells. In the third patient, who was treated with ultrasound-guided percutaneous injection of CAR-NK cells in the liver, a rapid tumor regression was detected in the injection site, demonstrating the direct effects of CAR-NK cells on metastatic colorectal cancer. Treatment efficacy was further supported by a loss of NKG2DL expression in biopsy samples collected from the injection site [11]. Importantly, NKG2D-based CARs have the potential to recognize NKG2D ligands expressed on immunosuppressive cells in the tumor microenvironment. Indeed, in a mouse model of ovarian cancer, NKG2D-based CAR-T cells were able to reduce immunosuppressive cells and induce activation of host antitumor immune cells both in early and established tumors. This suggests that regardless of the increased prevalence of immunosuppressive cells in established tumors, NKG2D-based CAR-cells may have the potential to induce immune responses in patients with early or late stage tumors and to recruit and activate antitumor immune cells with ensuing production of inflammatory cytokines [85].

NKG2D-based CARs can recognize NKG2D ligands expressed on several human tumor types, but these ligands are also expressed in some immune-mediated diseases such as rheumatoid arthritis, in celiac and inflammatory bowel diseases and in multiple sclerosis, which raises concerns about “on-target off-tumor” toxicity [86]. However, large numbers of activated lymphocytes, that express NKG2D and recognize NKG2D ligand expressing cells, have been infused into patients with little systemic toxicity [87,88,89,90].

In view of the flexibility of CAR-NK cell engineering, a NKG2D-based CAR-NK cell immunotherapeutic approach can also be envisioned for treatment of HCC.

## 5. NKG2DL-Specific Antibodies for NK Cell-Mediated ADCC

Human peripheral blood NK cells are broadly defined as CD3–CD56^+^ lymphocytes. They can be further divided into two subsets on the basis of their surface expression levels of CD56. CD56^bright^ NK cells have principally immunoregulatory properties mediated by a potent cytokine producing capacity, while CD56^dim^ NK cells predominantly display cytotoxic function. CD56^dim^ NK cells also express high levels of the low-affinity Fcγ receptor FcγRIIIA/CD16a, which allows recognition of antibody-coated target cells, inducing ADCC and cytokine production [91]. Genotypic variations of the FcγRIIIA receptor influence cell surface CD16a expression, antibody binding as well as ADCC activity, resulting in variable efficiency of monoclonal antibody (mAb) therapy [92]. In addition, immunoglobulin isotypes IgG1 and IgG3 exhibit high affinity for CD16a, as well as fucosylation and glycosylation patterns conferring variable affinity for CD16a, which directly correlate with their ability to trigger NK cell-mediated ADCC [93,94,95]. Such features (patient FcγRIIIA genotype and antibody Fc backbone) create the chance to optimize therapeutic treatment options.

So far, several ADCC therapies have been assessed in clinical trials including anti-CD20 mAb (non-Hodgkin’s lymphoma, chronic lymphocytic lymphoma), anti-ganglioside D2 mAb (neuroblastoma, melanoma), anti-human epidermal growth factor mAb (breast and gastric cancers), anti-epidermal growth factor receptor mAb (colorectal and head and neck cancer), and in several other tumors [96,97,98,99,100]. Nakano et al. successfully generated a chimeric anti-glypican 3 (GPC3) mAb able to induce not only ADCC against GPC3-positive human HCC cells but it was also efficacious against a Huh-7 human HCC xenograft [101]. Another study demonstrated that an anti-epidermal growth factor receptor variant III (EGFRvIII) mAb significantly suppressed tumor proliferation and angiogenesis in an HCC xenograft model [102]. Moreover, a phase I clinical trial showed that the humanized antiendoglin mAb TRC105, combined with sorafenib provided objective, relatively durable, responses in a proportion of patients with advanced HCC [103].

Recently, Ferrari de Andrade et al, presented an elegant approach to improve NK cell recognition of tumor cells, circumventing tumor immune escape and efficiently targeting NK cells to tumors [104]. In that study, a monoclonal antibody was selected that masks MICA and MICB α3 domain, the site of proteolytic shedding by metzincins, namely ADAMs, preventing loss of cell surface MICA and MICB by human cancer cells. Indeed, MICA/B α3 domain–specific mAb significantly increased the density of the stimulatory MICA and MICB ligands on the surface of tumor cells, reduced MICA shedding, and induced NK cell–mediated tumor immunity. Therefore, this mAb could be used in combination with established therapies that enhance MICA/B expression or in combination with other immunotherapies to activate NK cells and enhance antitumor immunity [105]. Moreover, the MICA/B α3 domain–specific mAb could also be engineered into NK cells for adoptive cell transfer to boost efficient tumor cell targeting, with no toxic off-target cell killing.

More recently, beside the traditional mAbs, bispecific killer cell engagers (BiKEs) have generated exciting expectations. BiKEs comprise 2 antibody fragments (scFvs), one recognizing a tumor antigen (e.g., CD19, CD20, CD33) and a second directed against CD16a on NK cells. This approach brings the cancer and NK cells jointly, enabling the formation of an immunological synapse and allowing NK cells to specifically and effectively promote their cytolytic function. More scFvs and IL-15 were further integrated to create tri- and tetra-specific killer cell engagers (TriKEs and TetraKEs) in order to increase therapeutic benefits by targeting more tumor antigens and boost NK cell responses [106,107]. Chan et al. created a BiKE containing an anti-CS1scFv and an anti-NKG2D scFv (CS1-NKG2D) that displayed a dose-dependent increase in specific cytotoxicity of NK cells as well as cytokine production in vitro, and significantly prolonged survival in a human multiple myeloma model [108]. In conclusion, BiKEs and TriKEs provide a non–cell-based immunotherapeutic approach that can harness the patients’ own NK cells against cancer. Clinical trials will determine their safety and efficacy in patients.

## 6. Conclusions

Cancer immunotherapy represents an epochal change in the oncological treatment landscape. While most studies focused on rescuing T-cells from exhaustion in an effort to unleash tumor-specific immune responses, it has become clear that NK cells offer several assets to be exploited for immunotherapy, with potentially less adverse effects. Based on its powerful antitumor immune response capabilities, NK cell immunotherapy has gradually been applied in clinical practice for treatment of cancer patients. Indeed, NK cells are one of the promising candidates in the development of advanced cancer immunotherapies, although very few clinical trials are currently exploring NK cells as a therapeutic option for HCC, either directly or after appropriate manipulation. The evidence reviewed above suggests that interventions at the activating receptor/ligand axes and/or cytokine (IL-15) stimulation of effector NK cells may represent a novel and interesting approach. Moreover, as NK cells target tumor cells sensitized by mAbs, combining targeted therapy with mAbs specific for selected critical molecules expressed on tumor cells would also be a promising therapeutic strategy when NK cells are specifically activated. As chemotherapy causes resistance in many types of cancer including HCC, applying NK cell immunotherapy either as a standalone treatment or in combination with chemotherapy would be valuable options in this setting.

## Figures and Tables

**Table 1:** Clinical trials for treatment of HCC involving the use of NK cells. A www.ClinicalTrials.gov search was performed using the terms “hepatocellular carcinoma” and “natural killer cells” (last updated 25 February 2020). List of abbreviations: Obser, Observational; CIK, cytokine-induced killer cell; IKC, Immune Killer Cells; TACE, Transcatheter Arterial Chemoembolization; NKT, Natural Killer T; HCC, Hepatocellular Carcinoma; NSCLC, Non-Small Cell Lung Cancer; SCLC, Small-cell Lung Cancer; BCLC, Barcelona clinic liver cancer; C, Completed; R, Recruiting; ANR, Active not recruiting; S, Suspended; U, Unknown.

ID	Study Phase	Clinical Trial	Interventions	Conditions	Status
NCT02854839	2	A Study of MG4101 (Allogeneic Natural Killer Cell) for Intermediate-stage of Hepatocellular Carcinoma	Biological: MG4101	HCC	C
NCT04162158	1 2	Safety and Efficacy of Allogeneic NK Cells Therapy in Patients With Advanced Hepatocellular Carcinoma	Biological: allogeneic NK cells therapy	HCC	R
NCT01147380	1	Safety Study of Liver Natural Killer Cell Therapy for Hepatoma Liver Transplantation (MIAMINK)	Biological: Liver NK cell inoculation	Liver Cirrhosis HCCE vidence of Liver Transplantation	C
NCT02008929	2	To Evaluate the Efficacy and Safety of MG4101(Ex Vivo Expanded Allogeneic NK Cell) (MG4101)	Biological: MG4101	HCC	C
NCT00769106	3	Study of Cytokine-induced Killer Cell (CIK) Treatment in Patients After Resection of Liver Cancer (HCC-CIK)	Biological: CIK treatment	HCC	C
NCT03515252	1	Study of Autologous Immune Killer Cells in Patients With Late Stage Hepatocellular Carcinoma or Lung Cancer	Biological: IKC	NSCLC Stage IIIB NSCLC Stage IV HCC by BCLC Stage Lung Cancer Liver Cancer	C
NCT03592706	2 3	Autologous Immune Killer Cells to Treat Liver Cancer Patients as an Adjunct Therapy	Biological: IKC Procedure: TACE	HCC Liver Cancer	R
NCT01749865	3	CIK Treatment for HCC Patient Underwent Radical Resection	Biological: CIK	HCC	C
NCT04106167	Obser	Long-term, Non-interventional, Observational Study Following Treatment With Fate Therapeutics FT500 Cellular Immunotherapy	Genetic: Allogeneic NK cell No study drug is administered in this study. Subjects who received an allogeneic, iPSC-derived NK cell in a previous trial will be evaluated in this trial for long-term safety and efficacy	Advanced Solid Tumor Lymphoma Gastric Cancer Colorectal Cancer Head and Neck Cancer Squamous Cell Carcinoma EGFR Positive Solid Tumor HER2-positive Breast Cancer HCC SCLC Renal Cell Carcinoma Pancreas Cancer Melanoma NSCLC Urothelial Carcinoma Cervical Cancer Microsatellite Instability Merkel Cell Carcinoma	R
NCT03008343	1 2	Combination of Irreversible Electroporation and NK Immunotherapy for Recurrent Liver Cancer	Device: Irreversible Electroporation Biological: NK	Recurrent Liver Carcinoma	C
NCT03841110	1	FT500 as Monotherapy and in Combination With Immune Checkpoint Inhibitors in Subjects With Advanced Solid Tumors	Drug: FT500 Drug: Nivolumab Drug: Pembrolizumab Drug: Atezolizumab Drug: Cyclophosphamide Drug: Fludarabine	Advanced Solid Tumor Lymphoma Gastric Cancer Colorectal Cancer Head and Neck Cancer Squamous Cell Carcinoma EGFR Positive Solid Tumor HER2-positive Breast Cancer HCC SCLC Renal Cell Carcinoma Pancreas Cancer Melanoma NSCLC Urothelial Carcinoma Cervical Cancer Microsatellite Instability Merkel Cell Carcinoma	R
NCT03319459	1	FATE-NK100 as Monotherapy and in Combination With Monoclonal Antibody in Subjects With Advanced Solid Tumors	FATE-NK100 as Monotherapy and in Combination With Monoclonal Antibody in Subjects With Advanced Solid Tumors	HER2 Positive Gastric Cancer Colorectal Cancer Head and Neck Squamous Cell Carcinoma EGFR Positive Solid Tumor Advanced Solid Tumors HER2-positive Breast Cancer HCC NSCLC Renal Cell Carcinoma Pancreatic Cancer Melanoma	A
NCT00909558	1	Safety and Effectiveness Study of Autologous Natural Killer and Natural Killer T Cells on Cancer	Biological: Autologous NK/NKT Cell Immunotherapy	Breast Cancer Glioma HCC Squamous Cell Lung Cancer Pancreatic Cancer Colon Cancer Prostate Cancer	S
NCT02725996	2	By Using Adoptive Transfer of Autologous NK Cells to Prevent Recurrence of Hepatocellular Carcinoma After Curative Therapy	Biological: NK cells Other: Curative therapy	HCC	U
NCT02399735	1	Safety Study of NK Cells From Sibship to Treat the Recurrence of HCC After Liver Transplantation	Biological: Low Dose NK cells ×4 times Biological: Normal Dose NK cells ×4 times Biological: Normal Dose NK cells ×8 times	HCC	U
NCT02839954	1 2	CAR-pNK Cell Immunotherapy in MUC1 Positive Relapsed or Refractory Solid Tumor	Biological: anti-MUC1 CAR-pNK cells	HCC NSCLC Pancreatic Carcinoma Triple-Negative Invasive Breast Carcinoma Malignant Glioma of Brain Colorectal Carcinoma Gastric Carcinoma	U
